# Puerarin Attenuated Early Diabetic Kidney Injury through Down-Regulation of Matrix Metalloproteinase 9 in Streptozotocin-Induced Diabetic Rats

**DOI:** 10.1371/journal.pone.0085690

**Published:** 2014-01-15

**Authors:** Yifei Zhong, Xianwen Zhang, Xianfan Cai, Ke Wang, Yiping Chen, Yueyi Deng

**Affiliations:** 1 Division of Nephrology, Longhua Hospital, Shanghai University of Traditional Chinese Medicine, Shanghai, China; 2 National Engineering Research Center for Biochip at Shanghai, Shanghai, China; University of Kentucky, United States of America

## Abstract

Radix puerariae, a traditional Chinese herbal medication, has been used successfully to treat patients with early stage of diabetic nephropathy. However, the underlined mechanism of this renal protective effect has not been determined. In the current study, we investigated the effects and the mechanism of puerarin in Streptozotocin (STZ)-induced diabetic rats. We treated STZ-rats with either puerarin or losartan, an angiotensin II receptor blocker, as compared to those treated with vehicle. We found that both puerarin and losartan attenuated kidney hypertrophy, mesangial expansion, proteinuria, and podocyte foot process effacement in STZ rats. In addition, both puerarin and losartan increased expression of podocyte slit diaphragm proteins such as nephrin and podocin. Interestingly, we found that puerarin treatment induced a more pronounced suppression of oxidative stress production and S-nitrosylation of proteins in the diabetic kidneys as compared to losartan treatment. Furthermore, we found that matrix metalloproteinase-9 (MMP-9), which is known to be activated by oxidative stress and S-nitrosylation of proteins, was also suppressed more extensively by puerarin than losartan. In conclusion, these data provide for the first time the potential mechanism to support the use of puerarin in the treatment of early diabetic nephropathy.

## Introduction

Diabetic nephropathy (DN) is one of the most common microvascular complications of diabetes as well as one of the most common causes of death in patients with diabetes [1]. Hyperglycemia control, blood pressure control, and use of ACE inhibitors (ACEI) or angiotensin receptor blockers (ARBs) are the gold standard treatment for patients with DN [2], [3], [4]. However, the current regime provides only partial protective effects and the incidence/prevalence of DN remains high. Therefore, it is critical to develop more effective treatment regimens for patients with DN.

Clinically, renal involvement in patients with diabetes is defined by the appearance of microalbuminuria at the early stage and then the progression to ESRD over several years. Microalbuminuria is considered as a marker for progression of DN to ESRD [5] [6]. Podocyte injury is a major factor contributing to the microalbuminuria in patients with DN [7]. Podocytes are the last glomerular barrier to prevent loss of protein in the urine. Of all glomerular morphological characteristics, the reduction in podocyte density is the strongest predictor of progressive DN [8] [9]. Therefore, treatment targeting on podocyte injury would be an important approach to prevent albuminuria and slow the progression of DKD. However, the exact cause of podocyte loss in DN remains unclear.

Although exact mechanisms of DN are still yet to be elucidated, previous studies suggest that the polyol pathway [10], the formation of advanced glycation end products [11], the hyperglycemia-induced activation of protein kinase C isoforms [12], and the hexosamine pathway [13] are major mechanisms mediating the development of DN. All these pathways are closely related to oxidative stress or elevated levels of reactive oxygen species (ROS). ROS is known to cause podocyte injury [14]. Over-production of ROS in podocytes has been shown to activate ERK1/2 signal pathway, leading to increased expression of matrix metalloproteinase-9 (MMP-9) [15]. S-nitrosylation of proteins, a marker of oxidative stress, is found to be increased in the early stage of diabetes [16]. It is known that S-Nitrosylation activates MMP-9 and induces neuronal apoptosis [17]. MMP-9 is a key enzyme to degrade type IV collagen, which is a major component of the glomerular basement membrane (GBM). Over-expression of MMP9 could alter GBM components thereby causing podocyte structural changes or detachment from GBM [18]. MMP-9 is also known to cleave podocalyxin in podocytes, which is a charge barrier to prevent microalbuminuria [19]. It has been shown that high glucose and TGF-β stimulated MMP-9 expression in cultured podocytes [15] [20] [21]. MMP-9 expression was shown to be increased in stressed podocyte and associated with the activity of integrin-linked kinase (ILK), a key kinase involved in podocyte adhesion to the GBM [22]. Interestingly, it was reported that the urinary podocyte number is correlated with plasma MMP-9 level in patients with chronic kidney disease [23]. A genetic association between MMP-9 polymorphisms and DN has been also reported [24]. Taken together, these studies support a key role of MMP-9 in podocyte injury and glomerular disease such as DN.

Traditional Chinese herbal medications have been used widely in China to treat patients with DN. However, mechanisms by which these herbal medications improve DN remain unclear. We have reported that Chen's Tangshen decoction, in which radix puerariae is a major component, is able to reduce microalbuminuria significantly in patients with early DN [25]. Moreover, puerarin, one of the active components of radix puerariae, exhibited strong anti-oxidative activity [26]. In addition, our previous studies suggest that expression MMP9 is increased significantly in glomeruli of early diabetic rats at day 7 after STZ-injection [27]. Therefore, we examined here whether puerarin can protect podocytes against injury and improve early DN in STZ-induced diabetic rats through inhibition of ROS production and suppression of MMP-9 expression, as compared to the rats treated with losartan, an angiotensin II receptor blocker, which is a standard therapy for DN.

## Materials and Methods

### Materials

Losartan was purchased from Merck & Co Inc (H20000371). Puerarin was purchased from NANJING CHIA TAI TIANQING (06060521). All other chemicals and reagents were purchased from Sigma-Aldrich, Shanghai, China. Antibodies against GAPDH (ab37168), nephrin (rabbit, ab58968), podocin (rabbit, ab93650), and MMP-9 (rabbit, ab7299) were purchased from Abcam, Cambridge, UK. Anti-S-Nitroso-Cysteine antibody is obtained from Sigma-Aldrich. Antibody for type IV collagen was obtained from Chemicon. Horseradish Peroxidase (HRP) conjugated goat anti-rabbit secondary antibody (ab6721) was also purchased from the Abcam.

### Animal and experiment design

Forty Wistar male rats (180±10 gram) were purchased from Shanghai SLAC Laboratory Animal Co. Ltd. (China). All rats were housed at the SPF certified animal facility in the Experimental Animal Center of the Shanghai University of Traditional Chinese Medicine and the room was at 21–25°C and 40–50% humidity under a 12-h light/dark cycle. All animals were given free access to standard rat chow and water. The animal protocols were approved by animal ethnic committee of the Shanghai University of Traditional Chinese Medicine. Diabetes was induced by injection of streptozotocin (STZ) as described previously [28]. Briefly rats were injected with 50 mg/kg body weight (BW) of STZ (dissolved in 10 mM sodium citrate buffer) once intraperitoneally (ip). After 72 hours of STZ injection, blood and urinary glucose levels were measured to determine successful rate of diabetic model creation. When blood glucose level was higher than 16 mmol/L and urinary glucose level was +++ to ++++, diabetic model was considered successfully created. For normal control, rats were injected with 10 mM sodium citrate buffer once ip. Rats were then randomly divided into four groups: 1) normal control (10 rats) with ip injection of water and gavage of water; 2) STZ induced diabetic model (10 rats) with ip injection of water and gavage of water; 3) STZ induced diabetic model (10 rats) with ip injection of puerarin (100 mg/kg BW) and gavage of water; 4) STZ induced diabetic model (10 rats) with ip injection of water and gavage of losartan (80 mg/kg BW). The dosage of puerarin was selected based on our previous studies [27]. The treatment was given daily for 7 days. Blood glucose level was determined in diabetic rats daily by using Roche glucometer and respective glucose strip.

### Determination of serum albumin

Blood samples were collected from rats at time of sacrifice from the abdominal aorta. Blood were left at room temperature for 1 h and 4°C for 6 h. Blood samples were then centrifuged at 2500 rpm for 15 minutes at 4°C. The supernatant was then stored at −20°C. Serum albumin was determined by an automatic clinical chemistry analyzer (COBAS INTEGRA® 800 Roche).

### Urinary protein determination

One day before sacrificing rats, rats were housed in the metabolic cages without food but with free access of water. Twenty-four hours urinary samples were collected and water intake was calculated. After urine collection, urine volume was recorded and 4 ml of urine were centrifuged at 2000 rpm for 10 minute. The supernatant were collected and kept at −20°C until albumin determination by an ELISA assay (Abnova, Taiwan, Catalog # : KA0501).

### Collection of kidneys and immunostaining for MMP-9, S-Nitrosylation of proteins, and type IV collagen

When rats were sacrificed, body weight was recorded and rats were anesthetized by ip injection of 1% pentobarbital (45 mg/kg BW). After anesthesia, abdomen cavity was opened to collect blood from abdominal aorta and the kidney was flushed with saline to remove blood. The kidneys were then removed. The left kidney was immediately frozen in −80 freezer for real time reverse transcriptase polymerase chain reaction (RT-PCR) and western blot analyses respectively. Part of the right kidney was snap-frozen in n-hexane cooled to −70°C and then 4 mm-thick sections were cut with a cryostat (Leica, CM1850, from Germany). After washing three times with PBS, the sections were incubated with 3% BSA for 30 minutes to block non-specific binding. The primary antibodies were incubated with the kidney sections overnight at 4°C. The PBS was used for negative control. After washing three times with PBS of 10 minutes each time, HRP conjugated goat anti-rabbit secondary antibody at dilution of 1∶1000 or 1∶2000 was incubated with the sections for 1 hour. After addition of reaction substance, the sections were then dried, sealed and stored at 4°C until observation under microscopy (Leica) with 100× objective.

### Electron microscope analysis of the kidney podocytes foot processes

Part of the right kidney was fixed in 3% glutaraldehyde for 2 hours and washed with PBS three times. Tissues were further fixed in 10% osmium tetroxide for 2 hours and then embedded in Epon812. After ultracut, sections about 50 nm thick were stained with lead citrate, ultrastructure (glomerulus basement membrane and podocyte processes) of the kidney was observed under Hitachi H-600 electron microscope (Hitachi Company, Japan) at magnification of 3,000× and 5,000× respectively. Thirty visions were observed for each section to insure that 100 vision fields were obtained for each rat. Quantification of podocyte effacement was performed as previously described [29]. Briefly, negatives were digitized, and images with a final magnitude of approximately X15,000 were obtained. ImageJ 1.26 t software (National Institute of Health, rsb.info.nih.gov/ij) was used to measure the length of the peripheral GBM and the number of slit pores overlying this GBM length was counted. The arithmetic mean of the foot process width (W_FP_) was calculated as the following:
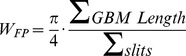
(Σslits indicates the total number of slits counted, ΣGBM length indicates the total GBM length measured in one glomerulus, and 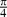 is the correction factor for the random orientation by which the foot processes were sectioned) [29].

### Real time reverse-transcriptase polymerase chain reaction (RT-PCR)

Total RNA was isolated from the left kidney by using Trizol reagent. One microgram of total RNA was used for the first strand cDNA synthesis by the Advantage PCR kit according to manufacturer's manual. Real time PCR was performed by the ABI Prism 7300 instrument with SDS software. The PCR primers for MMP-9, nephrin and podocin were designed by Anda Gene Company (Guangzhou, Guangdong province, China) and were listed in Table 1. One tenth of the first strand cDNA was employed for real-time RT-PCR of MMP-9, nephrin and podocin respectively. The following protocol was employed: after an initial denaturation step of 95°C for 10 minutes, amplification at 40 cycles of 15 seconds denaturation at 95°C, 60 seconds annealing at temperature as indicated in Table 1 for each gene and 60 seconds extension at 72°C. Fluorescent intensity was determined at the end of extension. The amount of mRNA was calculated by subtracting the value of GAPDH with the formulation of ΔCT = CT_sample_-CT_GAPDH_.

**Table 1:** Primers and conditions of real time polymerase chain reaction for rat genes.

Genes	Primers		Tm (°C)	Product (bp)
MMP-9	Forward	5′- CACTGTAACTGGGGGCAACT-3′	54.00	150
	Reverse	5′- CACTTCTTGTCAGCGTCGAA-3′	52.88	
Nephrin	Forward	5′- CAGCCTCTTGACCATCGCTAA-3′	54.31	168
	Reverse	5′- TGGTGGCCGTGCATTTG -3′	52.89	
Podocin	Forward	5′- TGGAAGCTGAGGCACAAAGA-3′	53.63	154
	Reverse	5′- CCCCTTCGGCAGCAATC -3′	52.41	
GAPDH	Forward	5′- GGCATTGCTCTCAATGACAA-3′	50.94	223
	Reverse	5′- TGTGAGGGAGATGCTCAGTG-3′	53.25	

### Western blot analysis

Total cellular protein was isolated by cellular protein extraction solution (1 X = 50 mM Tris pH 8.0, 0.5 mM EDTA, 150 mM NaCl, 0.5% NP-40 and 1X protease inhibitor cocktail (Sigma-Aldrich, Shanghai, China). Protein concentrations were measured by the Bradford method [30]. 30 mg total cellular protein were then mixed with 4X gel loading buffer, separated on 12% sodium dodecyl sulfate-polyacrylamide (SDS-polyacrylamide) gel under reducing conditions, and transferred onto Nitroplus-2000 membrane (Micron Separations Inc. Westborough, MA). Nonspecific antibody binding was blocked by pre-incubation of the membranes in 1X tris-buffered-saline (TBS) containing 5% skim milk for 1 hour at room temperature. Membranes were incubated overnight at 4°C with antibodies against respective proteins at different dilutions for each specific antibody in 1X TBS containing 0.1% Tween-20 and 2% skim milk. After washing, they were incubated with donkey anti-rabbit IgG or rabbit anti-mouse IgG at 1∶1000 dilutions for 1 hour at room temperature. Bands were visualized by employing the enhanced chemiluminescence kit according to the manufacturer's instructions and exposed to X-ray film.

### 8-hydroxy-2′-deoxy-guanosine (8-OHdG) ELISA assay

8-OHdG was determined in the kidney cortex by an ELISA kit (Biotechnology Co., Ltd. Shanghai, China) according to the manufacturer's instruction. Briefly, DNA was extracted from kidney cortices and digested according to the manufacture's instruction. 50 µl of DNA sample from kidney tissue or standard were added to each well and mixed with 50 µl of reconstituted primary antibody. Plate was shaken and covered with adhesive strip and then incubated at 4°C for overnight. After incubation, the contents of the wells were poured off and each well was washed with 250 µl of washing solution three times. Then 100 µl of constituted secondary antibody was added to each well and then the plate was shaken, covered with adhesive strip, incubated for 1 h at room temperature. After washing, a substrate solution was prepared and 100 µl of it was added to each well. The plate was incubated in the dark for 15 min at room temperature and then 100 µl of reaction terminating solution was added to each well. Absorbances were measured at 450 nm in a plate reader and standard curve was used to determine the amount of 8-OHdG in samples. Each sample was duplicated and value was expressed as optical density over mg of protein.

### Statistical analysis

To analyze differences between control and treatment groups, we performed the one-way ANOVA and Fisher's PLSD test as Post hoc test using SPSS (version 11.0) software (SAS Institute Inc. Cary, NC). Differences with *p* values below 0.05 were considered significant.

## Results

### Creation of diabetic rat model

**Figure 1:**
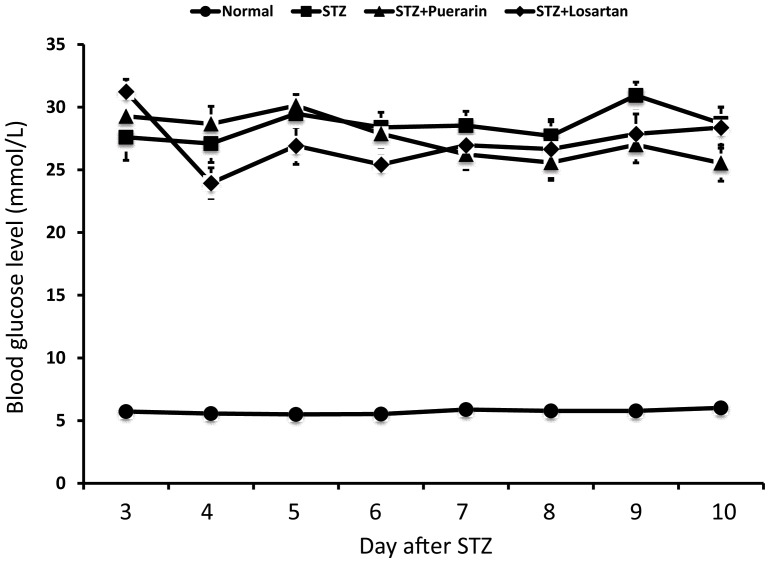
Effect of puerarin on blood glucose level of STZ-induced rats. Rats were injected with one dose of STZ and treated with or without puerarin for 7 days. Blood glucose level was examined once every day for 7 days. Data are presented as mean ± SE, n = 10.

The successful rate of diabetes induced by STZ in this study was 97.1% and only two rats died during treatment. The blood glucose level was shown in Figure 1. All diabetic rats had blood glucose level higher than 16.7 mmol/L. Blood glucose levels of STZ-rats fluctuated but did not differ significantly between diabetic rats treated with puerarin, losartan, or vehicle.

### Urinary proteinuria

**Figure 2:**
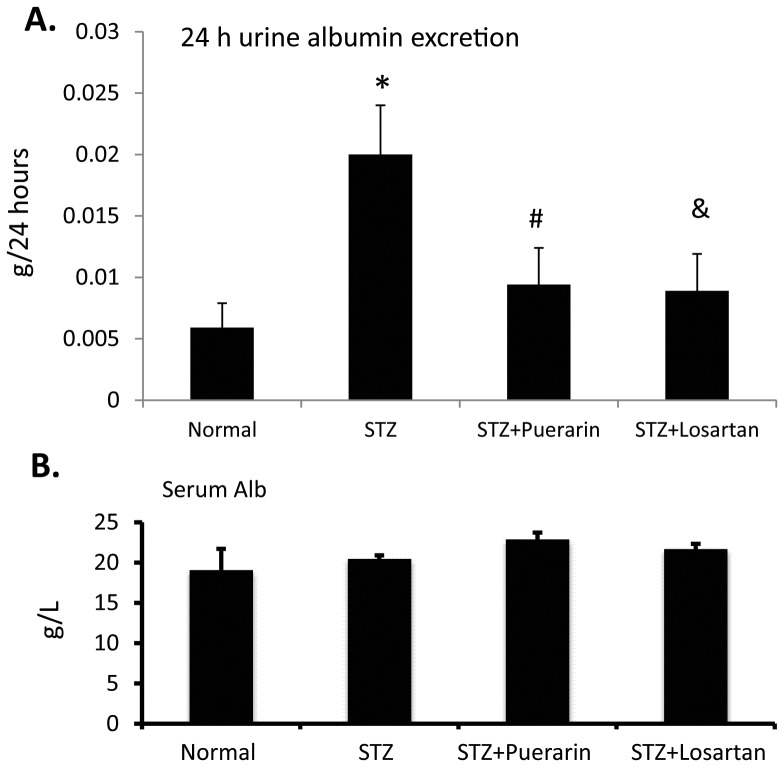
Blood and urinary albumin levels of STZ-induced rats. Bloods were obtained at the end of experiment while 24-h urine was obtained at the last day of experiment. **A.** Urinary albumin was determined by ELISA as described. **B.** Serum albumin was determined by an automatic clinical chemistry analyzer. Data are displayed as mean ± SE. *p<0.01 STZ rats compared to normal control, #p<0.05 STZ rats compared to STZ rats treated with puerarin, and ^&^p<0.05 STZ rats compared to STZ rats treated with losartan, n = 10.

As shown in Figure 2A, 24 hours urinary albumin excretion was increased significantly in diabetic rats as compared to non-diabetic control rats. Treatment of diabetic rats with either puerarin or losartan significantly reduced 24 hours urinary albumin excretion as compared to diabetic rats treated with vehicle. However, serum albumin level did not change among these groups (Figure 2B).

### Kidney histology

**Figure 3:**
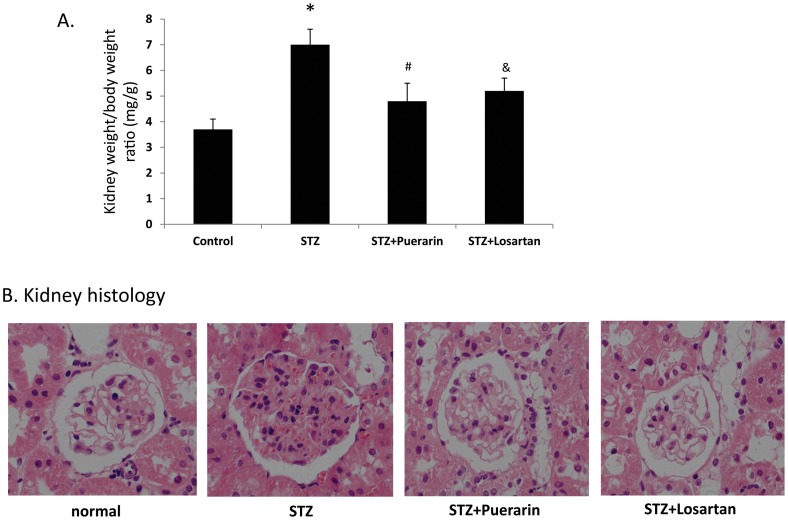
Kidney hypertrophy index and histology. **A.** Kidney and body weight of these rats were recorded and kidney hypertrophy was estimated by determination of kidney/body weight ratio. *p<0.01 STZ rats compared to normal control, #p<0.05 STZ rats compared to STZ rats treated with puerarin, and ^&^p<0.05 STZ rats compared to STZ rats treated with losartan, n = 10. **B.** Kidney histology was analyzed after H&E staining. The representative pictures are shown.

**Figure 4:**
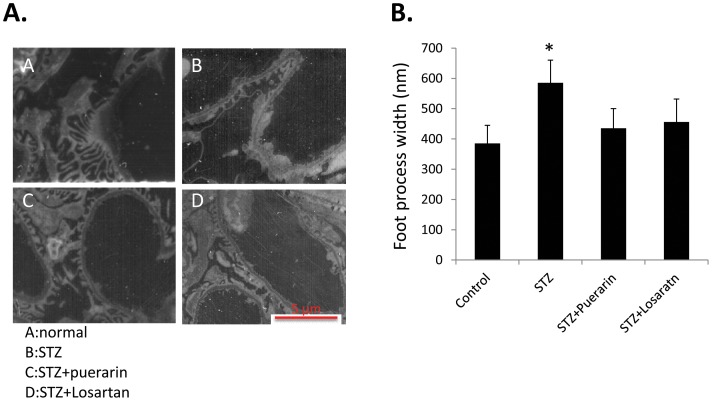
Kidney electron microscopy. **A.** Kidney samples from the four experimental groups were examined under transmission electron microscope as indicated. Panel A is for normal rats; panel B is for STZ rats, panel C is for STZ+puerarin rats and panel D is for STZ+losartan rats. Magnification 1,800×. **B.** Quantification of podocyte effacement is shown (n = 5, *p<0.001 STZ rats versus all other groups).

Kidney/body weight ratio was increased significantly in STZ rats. However, treatment of STZ rats with either puerarin or losartan prevented kidney hypertrophy (Figure 3A). Under light microscopy, there was an increase of in mesangial area in diabetic rats as compared to non-diabetic rats (Figure 3B). Under electron microscope, the kidney of STZ diabetic rats showed foot process effacement (Figure 4A). However, in rats treated with puerarin or losartan, the kidney ultra-structure appeared quite normal exception of significantly less foot process effacement as determined by quantification of the foot process width (Figure 4B).

### Expression of podocyte slit diaphragm markers

**Figure 5:**
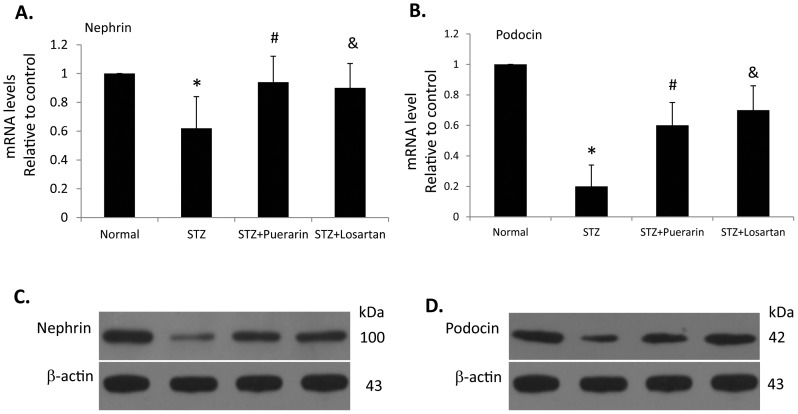
Expression of nephrin and podocin in the kidney. Total RNA and protein were extracted from the kidney respectively. Total RNA was subjected to real-time RT-PCR analysis (**A, B**) and protein was used for western blot analysis (**C, D**) for nephrin (**A, C**) and podocin (**B, D**). GAPDH was used as loading control of mRNA levels in real-time PCR and β-actin was used as loading control protein loading for western blot. Data are presented as mean ± SE. *p<0.05 STZ rats compared to normal control, #p<0.05 STZ rats compared to STZ rats treated with puerarin, and ^&^p<0.05 STZ rats compared to STZ rats treated with losartan, n = 5.

We then examined the expression of podocyte proteins that are involved in slit diaphragm. As shown by both real-time PCR and western blot, there was significant reduction of nephrin and podocin (p<0.05) in kidneys of STZ diabetic rats as compared to kidneys from control rats (Figure 5A, B, C, D). Treatment of STZ rats with either puerarin or losartan restored the normal expression of nephrin and podocin in diabetic kidneys (Figure 5A, B, C, D).

### ROS production

**Figure 6:**
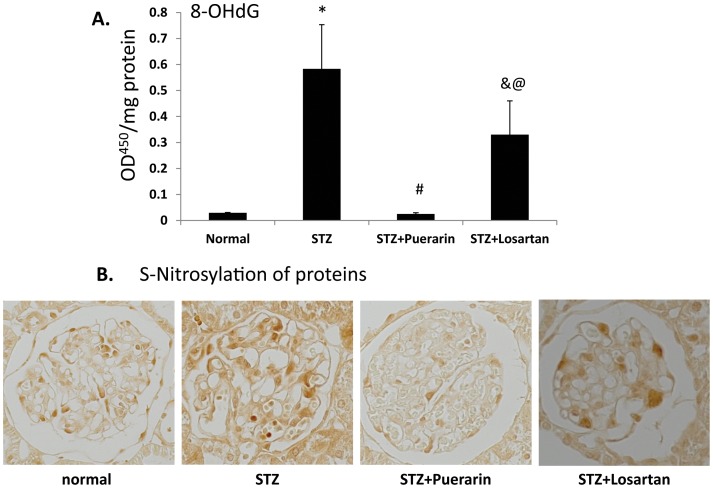
Assessment of oxidative stress in the kidney. **A.** 8-hydroxy-2′-deoxy-guanosine (8-OHdG) levels were determined in the kidney of these rats by ELISA assay as described in the methods and materials. Data are represented as mean ± SE, *p<0.001 STZ rats compared to normal control rats, #p<0.01 STZ rats compared to STZ rats treated with puerarin, ^&^p<0.01 STZ rats compared to STZ rats treated with losartan, and ^@^p<0.05 losartan treated STZ rats compared to puerarin treated STZ rats, n = 5. **B.** Immunostaining for S-Nitrosylation of proteins was performed in the kidney of these rats. The representative pictures of each group are shown.

We then investigated the potential mechanism that is responsible for podocyte injury in diabetic rats. As shown in Figure 6A, there was a dramatic increase of oxidative stress, as determined by 8-OHdG levels, in the kidney of diabetic rats as compared to normal rats. The 8-OHdG level was significantly suppressed in diabetic rats treated with puerarin but only a mild suppression was observed in diabetic rats treated with losartan. In addition, we performed immunostaining for S-nitrosylation of proteins, which is known to be induced by ROS, in kidney tissues of these rats. We found that there was an increased staining of S-nitrosylation of proteins in diabetic kidneys which was suppressed by treatment of puerarin but only a mild suppression was observed in losratan-treated rats (Figure 6B). These data suggest that puerarin induces a more significant suppression of oxidative stress in diabetic kidneys than losartan.

### Expression of MMP-9 and collagen IV

**Figure 7:**
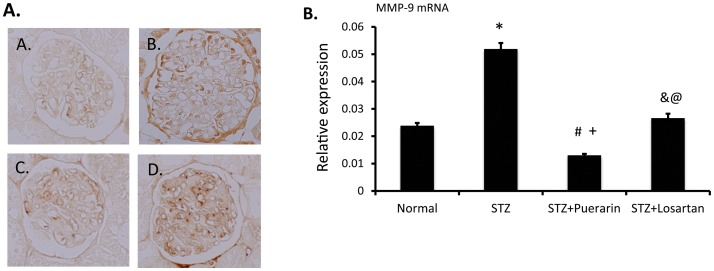
Expression of matrix metalloproteinase 9 (MMP-9) in the kidney. Kidneys from four experimental groups were collected for immunostaining and real-time PCR. **A.** Immunostaining of MMP-9 was performed. The representative pictures of each group are shown (a = normal, b = STZ, c = STZ+puerarin and d = STZ+losartan). **B.** MMP-9 mRNA levels were measured by real-time PCR in the kidney of these rats. Data are presented as mean ± SE, *p<0.01 STZ rats compared to normal control rats, #p<0.05 STZ rats compared to STZ rats treated with puerarin, ^&^p<0.05 STZ rats compared to STZ rats treated with losartan, and ^@^p<0.05 losartan treated STZ rats compared to puerarin treated STZ rats, #p<0.05 puerarin-treated STZ rats compared to normal control rats, n = 5.

**Figure 8:**
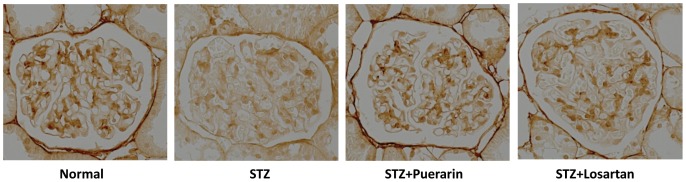
Immunostaining for type IV collagen. The representative pictures of each group are shown.

Since ROS and S-Nitrosylation of proteins are known to upregulate MMP-9 expression leading to the degradation of type IV collagen and podocyte injury [15], [18] [17] [19], we then examined MMP-9 mRNA and protein levels in the kidney of these rats. As shown in Figure 7A and 7B, both MMP-9 mRNA and protein levels were increased significantly in diabetic rats as compared to normal rats. Puerarin treatment significantly reduced both MMP-9 mRNA and protein levels in diabetic rats (p<0.05). Interesting, MMP-9 mRNA levels in puerarin-treated STZ rats were also lower than those in the normal control rats, while protein levels was not different. The inhibitory effect of losartan treatment on MMP-9 expression in diabetic rats was less remarkable than purerarin. These data suggest that puerarin and losartan reduces proteinuria and attenuates podocyte injury probably through different mechanisms. In addition, we found that the staining of collagen IV was reduced in diabetic kidneys (Figure 8). However, this was restored in kidneys of diabetic rats treated with puerarin and a partial restoration was also observed in losartan-treated rats.

## Discussions

Regardless of the current intervention with ACEI and ARBs, the prevalence of DN remains high. Therefore, there is urgent need to develop more effective therapy for DN. Unfortunately recent phase III clinical trials in patients with DN have all failed [31] [32]. Recent studies suggest that the incidence of diabetes and DN have been dramatically increasing in China over last decade [33] [34] due to changes of life style and dietary. Moreover, many patients seek traditional Chinese medicine (TCM) for treatment of DN in China. Extensive experience has been accumulated in this field by many physicians practicing TCM over years. We have reported that Chen's Tangshen decoction is one of the best herbal medicines, which can reduce microalbuminuria effectively in patients with early DN [25]. However, the mechanism of TCM in DN has not been well studied. Here, we explored renal protective mechanisms of puerarin, a major active component of Chen's Tangshen decoction, in STZ-induced diabetic rats. We showed that treatment of diabetic rats with puerarin effectively reduced proteinuria and podocyte foot process effacement, confirming the therapeutic value of Chen's Tangshen decoction. Since clinical data suggest that Chen's Tangshen decoction is effective in early DN, we selected to perform a short course of treatment in diabetic rats with early DN. However, in the future studies it would be interesting to determine whether puerarin could also improve kidney injury in animals or patients with more advanced DN and with other proteinuric kidney diseases. Based on the current study and our clinical experiences, puerarin is a safe drug and does not cause significant side effects. However, we don't know whether long-term treatment with puerarin could cause side effects. It is known that puerarin given intravenously could cause itching and nausea.

Albuminuria is a prognostic marker of DN [35]. The presence of albuminuria in patients with diabetes usually indicates underlined renal structural injury such as podocyte foot process effacement [36]. Moreover, ultra-structural changes of podocyte foot process may occur earlier than microalbuminuria [37]. In the current study, we observed significant change in the foot process of podocytes associated with proteinuria, which are both reversed after treatment with either puerarin or losartan. These data suggest that puerarin could be used to treat patients with early DN.

Puerarin is a component of the root of pueraria candollei wall of Leguminosae family. Puerarin, 7-hydroxy-3-(4-hydroxyphenyl)-1-benzopyran-4-one-8-β-D-glucopyranoside is one of the major isoflavonoid compounds from this plant [38]. Anti-oxidative activity of puerarin has been documented in cardiovascular [39] and neurological diseases [40]. However, the anti-oxidative function of puerarin has not been well studied in the kidney. Our study demonstrates for the first time that puerarin is able to reduce oxidative stress in diabetic animal model. It has been reported that several other active compounds extracted from the plants have anti-oxidant activity and these plants are often used to treat patients with kidney disease [41], [42], [43] [44], [45]. Therefore, we believe that reduction of oxidative stress may represent a common mechanism for herbal medications to improve kidney injury.

Since it is known that over-production of ROS induces MMP9 expression in kidneys [15] and MMP9 is known to cause podocyte injury in DN [18]. In addition, our previous study revealed a significant increase in MMP-9 expression in podocytes at 7 days after STZ injection [27]. These data suggest that MMP9 could be an important molecule mediating podocyte injury in early DN. Therefore, we examined the effects of puerarin on MMP9 expression in diabetic rats and we found that MMP9 is completely suppressed in kidneys of puerarin-treated rats. These data suggest that suppression of ROS-MMP9 pathway is one of the mechanisms that puerarin attenuates proteinuria and podocyte injury in diabetic rats.

MMP-9 is a member of a subfamily of MMP (the gelatinases – MMP2 & MMP-9) that share the ability to degrade basement membrane types IV and V collagens, elastin and gelatins [46]. Unlike MMP-2 that is constitutively expressed, MMP-9 has a restricted pattern of expression in developmental and adult tissues. Moreover its expression is also highly regulated by many cytokines and growth factors [18]. Because MMP-9 degrades type IV collagen in GBM, it could be anticipated that MMP-9 may play a pivotal role in renal development and glomerular disease. The role of MMP9 in kidney development has been well studied [47] [48], [49] [50]. However, the studies of MMP9 in glomerular disease are quite limited. It is known that MMP9 expression is induced in podocytes not only by ROS but also by high glucose and TGF-b [15] [20] [21]. MMP9 is also known to be associated with podocyte injury [19] [22] [23]. All these studies support a critical role of MMP9 in podocyte pathology and glomerular disease. In our study we found that there were significant increases in both oxidative stress and MMP-9 expression associated with reduced type IV collagen staining in the diabetic kidney at the early phase of DN. These findings suggest that ROS/MMP-9 is likely involved in the pathogenesis of early DN and suppression of ROS/MMP-9 might be a target to prevent the podocyte injury at the early phase of DN. Our current studies suggest that puerarin might be one of the drugs targeting ROS/MMP9 pathway to protect podocyte injury and improve DN. It should be noticed here that MMP9 is also known to play a role in kidney fibrosis through induction of epithelial-mesanchymal transition [51]. MMP9 is also involved in the recruitment of pro-inflammatory macrophages in experimental glomerulonephritis [52]. However, we believe that these roles of MMP9 are quite different from that we reported here in the early phase of DN.

We also found that puerarin had more pronounced anti-oxidative effects than losartan suggesting that puerarin may have different renal protective mechanisms than inhibitors of renin-angiotensin system. These findings suggest that puerarin could be used together with ACEI or ARBs to maximize renal protective effects in patients with early DN.

**Figure 9:**
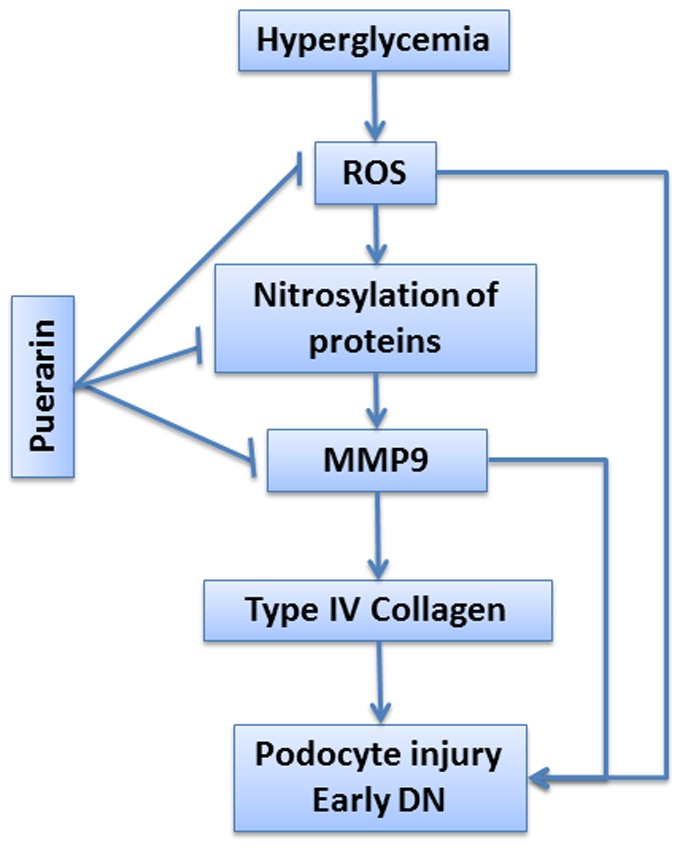
Summary schema. Diabetic milieu such as hyperglycemia induces ROS production and S-nitrosylation of proteins, which in turn activates MMP-9 expression. MMP-9 induces podocyte injury through alteration of GBM components such as degradation of type IV collagen. Puerarin prevents podocyte injury through attenuation of ROS production, inhibition of S-nitrosylation of proteins, and reduction of MMP-9 expression.

In conclusion, we demonstrated that puerarin attenuated proteinuria and podocyte injury in diabetic rats probable through reduction of oxidative stress and suppression of MMP-9 in the diabetic kidney as summarized in Figure 9. We provide here potential mechanism to explain why Chen's Tangshen decoction is effective in patients with early DN. Our data also suggest that puerarin, an active compound of Chen's Tangshen decoction could be developed as a potential drug to treat patients with early DN.
